# Nischarin regulates focal adhesion and Invadopodia formation in breast cancer cells

**DOI:** 10.1186/s12943-018-0764-6

**Published:** 2018-02-07

**Authors:** Mazvita Maziveyi, Shengli Dong, Somesh Baranwal, Suresh K. Alahari

**Affiliations:** 1 Department of Biochemistry and Molecular Biology, LSUHSC School of Medicine, New Orleans, LA 70112 USA; 2 Department of Biochemistry and Microbial Science, Central University of Punjab, Bathinda, 151001 India

**Keywords:** Nischarin, Invasion, Invadopodia, Migration, Focal adhesion, Breast cancer, Integrins

## Abstract

**Background:**

During metastasis, tumor cells move through the tracks of extracellular matrix (ECM). Focal adhesions (FAs) are the protein complexes that link the cell cytoskeleton to the ECM and their presence is necessary for cell attachment. The tumor suppressor Nischarin interacts with a number of signaling proteins such as Integrin α5, PAK1, LIMK1, LKB1, and Rac1 to prevent cancer cell migration. Although previous findings have shown that Nischarin exerts this migratory inhibition by interacting with other proteins, the effects of these interactions on the entire FA machinery are unknown.

**Methods:**

RT-PCR, Western Blotting, invadopodia assays, and immunofluorescence were used to examine FA gene expression and determine whether Nischarin affects cell attachment, as well as the proteins that regulate it.

**Results:**

Our data show that Nischarin prevents cell migration and invasion by altering the expression of key focal adhesion proteins. Furthermore, we have found that Nischarin-expressing cells have reduced ability to attach the ECM, which in turn leads to a decrease in invadopodia-mediated matrix degradation.

**Conclusions:**

These experiments demonstrate an important role of Nischarin in regulating cell attachment, which adds to our understanding of the early events of the metastatic process in breast cancer.

**Electronic supplementary material:**

The online version of this article (10.1186/s12943-018-0764-6) contains supplementary material, which is available to authorized users.

## Background

During metastasis, cancerous cells have to migrate through the extracellular matrix (ECM) for intravasation into blood and lymphatic vessels [1]. In order for this to occur, a cell must be able to detach from its original site and migrate through the breast stroma. The breast stroma, also known as the ECM, which is mostly composed of fat and matrix fibers while the rest is connective tissue [2]. This means a cell must be able to attach to ECM components through focal adhesions, as well as, degrade the ECM with matrix metalloproteinases. As the epithelial to mesenchymal (EMT) switch is in progress, the tumor has to rely on an altered microenvironment for migration [3]. Altered ECM composition and stiffness induce mammary malignancies [4–6]. This altered ECM is partly due to changes in fiber organization, and increased tension from cell-matrix adhesions [7, 8]. Since the ECM appears to be a key factor in breast cancer risk, it is important to study its regulation.

Nischarin is a cytosolic protein that binds to the cytosolic domain of integrin α5 to inhibit cell migration and invasion [9]. Nischarin interacts with Rac1, LIM Kinase (LIMK), Liver Kinase B1 (LKB1), p21 Protein (Cdc42/Rac)-Activated Kinase 1 (PAK1), Rab14 and Insulin Receptor Substrates 1–4 (IRS1–4) [9–13]. Nischarin’s location at chromosome 3p21.1 puts it in a category of genes that are associated with the development of many cancers. In vivo and in vitro studies from the Alahari lab have classified Nischarin as a tumor suppressor because it inhibits tumor progression and metastasis [11]. Since mutations in Nischarin are related to reduced patient survival [11], our goal is to understand how Nischarin affects cell interaction with the ECM. This study has determined that Nisch decreases the expression of several FA proteins, including Integrins. Furthermore, we have discovered that this aberrant cell attachment translates into an inability of these cells to form invadopodia and secrete matrix metalloproteinases. Since this work explains the role of Nischarin in the early stages of the metastatic process, the results of our research will contribute to our understanding of the tumor microenvironment. Understanding the role Nischarin and other tumor suppressors play in the prevention of breast cancer cell survival and migration will provide more knowledge for novel breast cancer therapies.

## Methods

### Cell culture

Human breast cancer MDA-MB-231, MDA-MB-231 Nischarin, MCF7 shScramble, MCF7 GFP Nisch, MCF7 shNisch, WT-Nischarin mouse embryonic fibroblasts (MEFs), HET-Nischarin MEFs, Null Nischarin MEFs, HEK293, Cos7, and NIH3T3 cells were grown in Dulbecco’s Modified Eagle Medium (DMEM) at 37 °C, 5% CO_2_ supplemented with 10% fetal bovine serum and 1% penicillin/streptomycin (Gibco, Waltham, MA). MCF10A cells were grown in DMEM/F12 media supplemented with 10% fetal bovine serum and 1% penicillin/streptomycin (Gibco, Waltham, MA). For wound healing assays the cells were seeded on a 6-well plate and allowed to attach for 12 h. Then, a wound was created with a 200 μl tip and imaging was done at various time points. Percentage of wound closure was obtained using Image J. For live cell imaging, different subsets of 10,000 MDA-MB-231 cells were plated onto gelatin-coated 35-mm glass bottom dishes (2D) (MatTek) or NIH3T3 fibroblast-derived matrices (3D) overnight. The cells were cultured at 37 °C with 5% CO2 using a Live Cell Environmental Chamber (NEUE Group, Ontario, NY). Five random acquisition points were pre-determined for each experimental well, each pre-set location was photographed every 10 min for a period of 10 h using an Olympus IX71 microscope. Cell position and average speed were determined and calculated using Slidebook software.

### Tissues used

Twenty human breast cancer surgical specimens, thirteen malignant and seven non-cancerous tissues were obtained as frozen tissue sections from the Southern Division (Birmingham, AL), Eastern Division (Philadelphia, PA), and Mid-Western Division (Columbus, OH) of the Cooperative Human Tissue Network.

### Real-time PCR

Total RNA was isolated from cultured cells with TRIzol reagent (Invitrogen, Carlsband, CA). cDNA was generated from 2μg of RNA using the Invitrogen/Applied Biosystems/ABl High Capacity cDNA Reverse Transcriptase Kit (Carlsband, CA). For gel detection, samples were run on a 3% agarose gel. Quantitative real-time PCR (qRT-PCR) was performed with 2× SYBR green mix (Roche, Basel, Switzerland) according to the manufacturer’s instructions. ΔCt was calculated as Ct (sample)-Ct (βactin or GAPDH). Relative expression was calculated as ∆∆Cq (2^-ΔCt(sample)/ 2^-ΔCt (control cell line).

### Transwell invasion assay

The invasion assays were performed using Tanswell invasion chambers (Corning, Corning, NY) coated with 10 μg/ml Fibronectin (bottom) and matrigel (top). MCF10A cells were transferred to the top of the chamber with the indicated media. After incubation for 24 h at 37 °C in an atmosphere containing 5% CO_2_, invaded cells on the lower surface were stained with crystal violet stain and counted under a light microscope.

### Coimmunoprecipitation

For Nischarin-Cortactin binding experiments, HEK293 cells were transiently transfected with 4 μg of Flag-Cortactin and Myc-Nischarin using Lipofectamine® Reagent 2000 (Invitrogen, Carlsband, CA). As a control, 3 μg Myc-βgal was cotransfected with Flag-Cortactin. Forty-eight hours later, cells were lysed in lysis buffer (50 mM Tris, pH 7.5, 0.1% Triton X-100, 0.3 M sucrose, 100 mM KCl, and 1 mM CaCl) with protease inhibitors (1 mM PMSF, 2 μg/ml aprotinin, and 5 μg/ml leupeptin). The lysates were immunoprecipitated with appropriate antibodies and pulled down by Protein G Sepharose beads (Uppsala, Sweden). Proteins were then immunoblotted for Myc and Flag antibodies.

### Antibodies

Antibodies and dilutions were used as follows: mouse monoclonal anti-Nischarin (BD Biosciences, San Diego, CA; 1:1000), rabbit polyclonal anti-human Fibronectin (Sigma, St. Louis, MO; 1:1000), rabbit polyclonal anti-Cortactin (Santa Cruz, Dallas, TX; 1:3000), rat anti-Flag (Stratagene, San Diego, CA; 1:5000), mouse monoclonal anti-human C-myc (BioXCell, West Lebanon, NH; 1:2500), mouse monoclonal anti-Paxillin (BD Biosciences, San Diego, CA; 1:5000), mouse monoclonal anti-HIC5 (BD Biosciences, San Diego, CA; 1:5000), mouse monoclonal anti-CRP1 (BD Biosciences, San Diego, CA; 1:1000), mouse monoclonal anti-MCAM (BD Biosciences, San Diego, CA; 1:1000), mouse monoclonal anti-Zyxin (Santa Cruz, Dallas, TX; 1:100), rabbit polyclonal anti-VASP (Sigma, St. Louis, MO; 1:100), rabbit polyclonal anti-Integrin α5 (EMD Millipore, Billerica, MA; 1:100) and mouse monoclonal anti-Vinculin (Sigma, St. Louis, MO; 1:5000). The SNAKA51 antibodies (1:100) were a gift from Kenneth Yamada.

### Fibroblast matrices

To prepare coverslips for matrix deposition, 2 ml of 0.2% gelatin was added to the coverslips and incubated for 1 h at 37 °C. The gelatin was aspirated and 2 ml of cold PBS was added to wash the coverslip. After aspiration of the PBS, 2 ml of 1% glutaraldehyde was added and incubated at 37 °C for 30 min. The coverslips were washed three times for 5 min each with cold PBS. 2 ml of 1 M ethanolamine was then added and incubated for 30 min at room temperature. The three PBS washes were repeated again to remove all traces of ethanolamine. 2 ml of matrix media (DMEM + 10% FBS + P/S + Anti-Anti +50μg/ml ascorbic acid) was then added. Cells were plated on the coverslips in 2 ml of matrix media. These cells were cultured for 24 h. After 24 h, the medium was aspirated and replaced with fresh matrix medium every day for six days. On day 6, the medium was carefully aspirated and the matrix was decellularized. Curvelet transform plus FIRE (CT-FIRE) algorithm was used to analyze fiber width and number.

### Fluorescent-gelatin degradation assay

Coverslips were cleaned with 20% nitric acid and coated with poly-L-lysine in a 24-well plate. The poly-L-lysine was fixed with 0.5% glutaraldehyde before adding the Oregon green Gelatin (Life Technologies, Calsbad, CA). Residual reactive groups were quenched by sodium borohydride. 4 X 10^3^ cells were plated and incubated at 37 °C. Images were visualized by confocal microscopy.

### Statistical analysis

All statistical analyses were performed using Graphpad Prism 5.0 software (San Diego, CA) using a nonparametric two-tailed *t* test or one way anova.

## Results

### Nischarin alters the expression of focal adhesion proteins

**Fig. 1:**
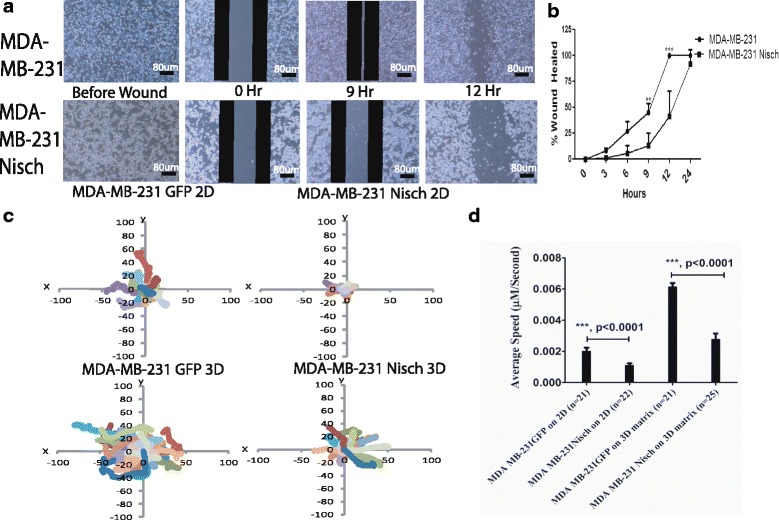
Nischarin Decreases Cancer Cell Migration. **a** Representative images of the wound healing assays performed on MDA-MB-231 and MDA-MB-231 Nisch cells at 0, 3, 6, 9, 12 and 24 h (*n* = 3 each) scale bar: 80uM. Figure shows images before wound, at 0, 9 and 12 h. **b** Quantitation of the wound healing assay’s with Image J. Stable transfection of Nischarin in MDA-MB-231 cells leads to a decrease in the percentage of wound closure. **c** Migration tracks of 231 and 231 Nisch cells on gelatin (2D) or 3D fibroblast-derived matrices. **d** Average speed of 231 and 231 Nisch cells on gelatin (2D) or 3D fibroblast-derived matrices. **p < 0.01 and ***p < 0.001

The tumor suppressor Nischarin has previously been shown to inhibit cell migration in breast cancer cells [14]. To confirm that Nischarin prevents cancer cell migration, we performed a time course wound healing assay with our previously published MDA-MB-231 and MDA-MB-231 Nischarin cells [11] for up to 24 h (Fig. 1a). Quantitation of the percentage of wound closure at 0, 3, 6, 9, 12, and 24 h revealed that MDA-MB-231 Nischarin cells have an overall reduced cell migration and the effect is more robust at 9 and 12 h (Fig. 1b). To further validate these findings, we explored whether Nischarin is still able to slow down the migration of cells attached in a 3D environment. We tracked live cell migration of cells seeded on a thin 2D gelatin layer or 3D fibroblast-derived matrices for a period of ten hours (Fig. 1c). MDA-MB-231 Nisch cells had a reduction in average speed on both 2D and 3D environments when compared to 231 cells (Fig. 1d). These experiments confirm our previously published work showing that Nischarin reduces cell migration.

**Fig. 2:**
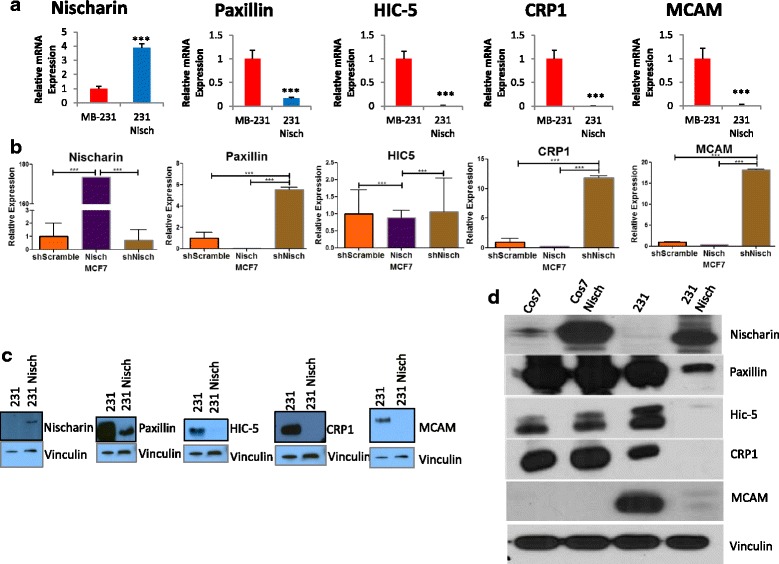
Nischarin Decreases FA Protein Expression in Cancer Cells. **a** Quantitative RTPCR (qRTPCR) of Nischarin, Paxillin, HIC-5, CRP1, and MCAM in MDA-MB-231 and MDA-MB-231 Nisch cells, as well as, **b** MCF7 scramble, MCF7 Nisch and MCF7 shNisch cells (*n* = 5 each). GAPDH was used as the internal control. **c** Western Blot of Nischarin, Paxillin, HIC-5, CRP1, and MCAM in 231, 231 Nisch cells. Vinculin is included as the loading control for the Western Blots. **d** Western Blot of Nischarin, Paxillin, HIC-5, CRP1, MCAM and Vinculin in Cos7 and 231 cells with or without Nischarin

Although previous findings showed that Nischarin exerts this migratory inhibition by interacting with other proteins, such as LIMK and PAK1 [11], the effects of these interactions on the entire FA machinery are unknown. FAs are the protein complexes that link the cell cytoskeleton to the ECM and their presence is necessary for cell attachment [15]. Tyrosine phosphorylation is a key event that occurs prior to the recruitment of other FA proteins [15]. To assess the effects of Nischarin on the FA machinery, we performed qRT-PCR and Western Blots of twelve key focal adhesion proteins. Out of twelve, we found five genes with consistently significant decreases in expression (Fig. 2). Paxillin is one of the first proteins to arrive at a nascent focal adhesion site [16]. Nischarin-expressing MDA-MB-231 and MCF7 cells have a significant reduction of Paxillin RNA and protein compared to Nisch-lacking cells (Fig. 2a-c). The ability of FA proteins to form protein-protein interactions is necessary for the formation of stable FAs. HIC-5 is a Paxillin-related protein that coordinates multiple protein-protein interactions in the early stages of FA development [15, 17]. MDA-MB-231 and MCF7 cells expressing Nisch have significantly less HIC-5 RNA and protein when compared to MDA-MB-231 cells (Fig. 2a-c).

In the intermediate stages of FA development, Actin filament bundling must occur in order for there to be a stable protrusion. Cysteine-rich protein 1 (CRP1) is a protein that regulates Actin filament bundling by binding to the Actin-bundling protein α-Actinin [18]. We also found reduced expression of these proteins in the presence of Nischarin (Fig. 2a-c). Finally, we assessed the expression of the Melanoma Cell Adhesion Molecule (MCAM), also known as, CD146, which is considered a marker for mesenchymal cells [19]. Nisch-expressing cells had a reduction in MCAM expression (Fig. 2a-c). To determine whether this decrease of FA protein expression is specific to cancer cells, we transiently transfected Cos7 cells (monkey kidney) with Myc or Myc-Nischarin and examined expression of the proteins. The presence of Nisch did not alter expression of any one of the FA proteins in Cos7 cells but only in the MDA-MB-231 cancer cells (Fig. 2d). These data demonstrate that the presence of Nischarin leads to a decrease in the expression of some FA proteins in cancer cells only.

### Nischarin alters integrin expression

**Fig. 3:**
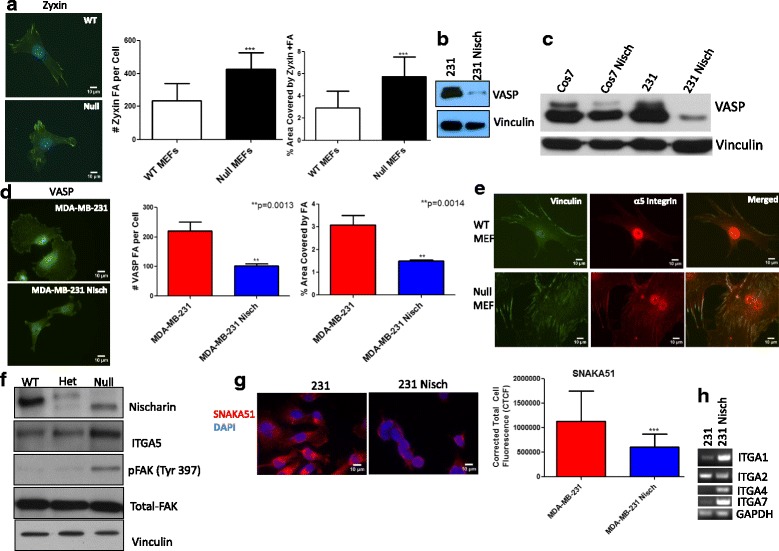
Nischarin Alters Integrin Expression. **a** WT (*n* = 20) and Null MEFs (n = 20) were seeded onto Fibronectin coated coverslips (10 μg/ml) overnight then stained with Zyxin and DAPI. # FA per cell and % area covered by FA was acquired using CellProfiler. **b** Western Blot detection of VASP in 231 and 231 Nisch cells. **c** Western Blot of VASP and Vinculn in Cos7 and 231 cells with or without Nischarin. **d** MDA-MB-231 (n = 3) and MDA-MB-231 Nis (n = 3) cells were seeded onto Fibronectin coated coverslip (10 μg/ml) overnight then stained with VASP. # FA per cell and % area covered by FA was acquired using CellProfiler. **e** WT and Null MEFs were seeded onto Fibronectin coated coverslips (10 μg/ml) overnight then stained with Vinculin (green) and α5 Integrin (red). **f** Western Blot of Nischarin, ITGA5, pFAK, t-FAK, and Vinculin in WT, HET and Null MEFs. **g** 231 (*n* = 50) and 231 Nis (*n* = 32) cells were seeded on a Fibronectin coated coverslip (10 μg/ml) overnight then stained with SNAKA51 and DAPI. Cell fluorescence was quantitated through Image J. Corrected Total Cell Fluorescence (CTCF) = Integrated Density – (area of selected cell x mean fluorescence). **h** RT PCR samples of ITGA1, 2, 4 and 7 RNA in 231 and 231 Nisch cells run on a 3% agarose gel. All scale bars indicate 10 μm

Zyxin is a protein that concentrates along the Actin cytoskeleton and localizes at FA sites [20]. We previously determined the number of FAs in Nisch positive and negative cells. Mouse embryonic fibroblasts (MEFs) are widely used for quality visualization of FAs due to their complete mesenchymal nature. We stained WT and Null Nisch MEFs [21] with Zyxin and quantitated the number of FAs using CellProfiler. Null Nischarin MEFs had an increase in the number of FAs and percentage of area covered by FAs (Fig. 3a). These quantitated images support the data in Fig. 1 that showed the decrease of migration and FA protein expression in Nischarin-expressing cells.

The Ena/VASP family of proteins play an important role in cell migration, invasion and adhesion [22]. In fact, they are capable of binding the Integrin α5 (ITGA5) cytoplasmic tail similar to Nischarin [22, 23]. We examined VASP protein expression and found that its expression is reduced in Nischarin-expressing MDA-MB-231 cells (Fig. 3b). We determined that this is cancer cell specific since addition of Nisch to Cos7 cells does not affect VASP expression (Fig. 3c). To further assess the impact of Nischarin on the number of VASP-positive FAs, we stained MDA-MB-231 and MDA-MB-231 Nisch cells with VASP and used the CellProfiler software to count the number of VASP FAs per cell (Fig. 3d). MDA-MB-231 Nisch cells had a reduction in the number of VASP FAs and percentage of area covered by VASP positive FAs. To determine whether we see the same pattern in WT and Null Nischarin MEFs, we stained them with VASP and quantitated FA numbers using Cell Profiler. Null Nischarin MEFs had an increase in the number of VASP FAs and percentage of area covered by VASP positive FAs (Additional file 1: Fig. S1). Since Nisch and VASP both bind to Integrins, we next determined whether Nischarin binds to VASP to reduce its presence in FAs. Co-Immunoprecipitation experiments between Nischarin and VASP showed that there is no interaction between the two proteins (Additional file 1: Fig. S1B). These results demonstrate that while Nischarin and VASP both bind ITGA5, they do not interact but the presence of Nisch leads to a decrease in VASP-positive FAs.

We have established that Nisch regulates FA protein expression. Integrins are membrane proteins that connect the FA protein complexes to the extracellular matrix (ECM) [15]. We have previously shown that Nischarin binds to and inhibits the expression of ITGA5 [23]. To support our previous findings, we visualized whether this Nisch-mediated ITGA5 reduction is still present in FA sites. Vinculin is often used as a marker to detect Integrin-mediated cell-matrix adhesions by immunofluorescence [24, 25]. Next, to determine whether Nischarin inhibits targeting of ITGA5 to FA sites, we stained WT and Null Nisch MEFs with Vinculin (green) and ITGA5 (red) (Fig. 3e). Merged images of Vinculin and ITGA5 showed less colocalization of ITGA5 with FAs in WT MEFs when compared to Null MEFs (Fig. 3e). We further validated the expression of ITGA5 and activation of its regulator, FAK. Western Blotting demonstrated an increase in protein expression of ITGA5 and pFAK in Null Nisch MEFs (Fig. 3f). Cancer cells exhibit increased autophosphorylation of FAK at Y397 [26]. This activated FAK is responsible for initiating the FA signaling pathway, thus allowing Integrin activation. We have established that Nisch inhibits ITGA5 expression, and thus the whole Integrin α5β1 complex is reduced [11]. For the Nisch blot, the lower band present in Null MEFs reflects the 172 amino acid deletion that produces a Null Nisch phenotype [21]. The doublet band for HET MEFs reflects the normal allele (top) and the truncated allele (bottom) [21].

Furthermore, we identified the levels of active Integrin α5β1 by immunofluorescence using the conformation-dependent ITGA5 antibody SNAKA51 [27]. There was significantly less SNAKA51 fluorescence in 231 Nis cells, indicating less active ITGA5 (Fig. 3g). This is the first time we have been able to quantify the reduction in the amount active Integrin α5β1.

Finally, we assessed whether Nischarin alters the expression of other Integrins. We performed RT-PCR experiments that showed alterations in the expression of four other Integrins in the presence of Nischarin. Integrins α1, 4, and 7 levels were increased, while Integrin α2 was decreased in the presence of Nischarin (Fig. 3h). Taken together, we have established that Nischarin regulates the expression of multiple α-Integrins.

### Nischarin regulates Invadopodia

**Fig. 4:**
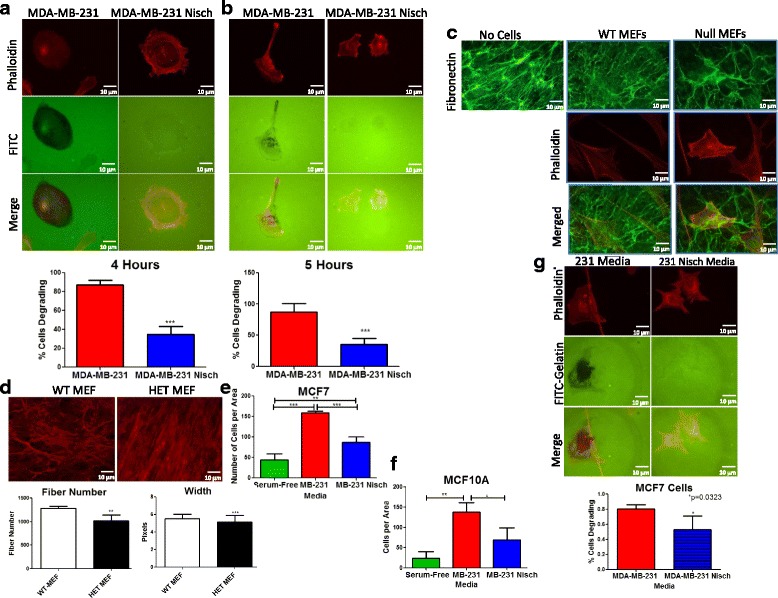
Nischarin Decreases Invadopodia Formation. **a** MDA-MB-231 (*n* = 70) and MDA-MB-231 Nisch cells (n = 20) were seeded on a fluorescent gelatin matrix (green) and allowed to degrade for 4 h. **b** 231 (*n* = 61) and 231 Nisch cells (*n* = 27) were seeded on a fluorescent gelatin matrix (green) and allowed to degrade for 5 h. Cells were then fixed and stained with Phalloidin (red) to visualize the cells degrading using the Nikon Eclipse Ti. The percentage of cells degrading was determined manually. All experiments were performed in triplicates. **c** WT and Null MEFs were seeded on decellularized NIH3T3 FDMs for 24 h. Cells were then fixed, stained with Fibronectin (green) and Phalloidin (red), then visualized using the Nikon Eclipse Ti. Mean fluorescence was determined by ImageJ. **d** Fibroblast-Derived Matrices (FDMs) were generated from WT (n = 5) and HET (n = 5) MEFs and fiber number (bottom left) and width (bottom right) were quantitated by CT-FIRE. **e** MCF7 and **f** MCF10A cells were seeded in an invasion chamber with either serum-free media or media from cultured 231 or 231 Nisch cells. 24 h later, invaded cells were manually counted after crystal violet staining. **g** Fluorescent gelatin matrix degradation of MCF7 cells cultured in media isolated from cultured 231 or 231 Nisch cells. The percentage of cells degraded was determined manually

Integrins also stabilize invadopodia, which are protrusive cell-matrix adhesions that contain proteases for ECM degradation. In addition to proteases, these protrusions are enriched with proteins including, Cortactin, Tks4 and Tks5 [28]. To determine whether Nischarin affects invadopodia formation, we performed a gelatin degradation assay [29]. To compare degradative patterns between MDA-MB-231 and MDA-MB-231 Nischarin cells, we plated the cells on fluorescent gelatin-coated coverslips and allowed them to degrade the gelatin matrix. A greater number of MDA-MB-231 cells degraded the fluorescent substrate at both four and five hours of time (Fig. 4a-b). To determine whether this degradation also occurs on Fibronectin fibers, we stimulated NIH3T3 fibroblasts to produce Fibronectin-derived matrix (FDM). After decellularization, we plated WT- Nisch and Null-Nisch MEFs on the NIH3T3 FDMs to visualize matrix alterations. Seeding Null MEFs on the FDMs reduced the Fibronectin signal (Fig. 4c), suggesting that the Null MEFs have degraded the matrix. Taken together, Nischarin negative (Null-Nisch) cells degrade the ECM at a greater rate than WT cells.

To further asses this in vitro while still maintaining a physiologically realistic culture substrate, we generated fibroblast-derived Fibronectin-rich matrices from WT-Nisch and HET-Nisch MEFs. Fibroblasts were cultured for eight days in the presence of ascorbic acid to stimulate matrix production. At day 8, cells were decellularized and Fibronectin was detected on the fibers by confocal microscopy (Fig. 4d). WT-Nisch and HET-Nisch fibronectin fibers were quantitated by CT-FIRE and the analysis revealed that WT-Nisch fibers have a greater fiber number and fiber width than the Het-Nisch fibers (Fig. 4d). These data suggest that the HET cells that were seeded on the substrate were degrading the matrix.

Since the invadopodia are enriched with several secreted proteins, we explored the effects of secreted media from MDA-MB-231 and MDA-MB-231 Nisch cells on the invasion of moderately invasive MCF7 cells. Media from MDA-MB-231 cells increased the invasiveness of MCF7 cells, while addition of media from MDA-MB-231 Nisch cells reduced the percentage of MCF7 cells degrading the fluorescent gelatin matrix (Fig. 4e). To further explore this on non-invasive cells, we isolated media from MDA-MB-231 and MDA-MB-231 Nisch cells and cultured them with normal MCF10A cells to monitor invasion. MCF10A cells are normal, noncancerous breast epithelial cells that typically do not have the capacity to invade. Adding media from MDA-MB-231 cells significantly induced the invasiveness of MCF10A cells in transwell inserts (Fig. 4f). Culturing the cells with media from MDA-MB-231 Nisch cells significantly reduced the invasive capability of the cells. Next, we wanted to determine whether media from MDA-MB-231 Nisch cells reduced invadopodia formation. Similar to the transwell invasion assays, MCF7 cells cultured in 231 Nisch media had a reduction in the percentage of cells degrading the gelatin matrix (Fig. 4g). Taken together, these results suggest that Nisch prevents invadopodia formation, and media isolated from Nisch expressing cancer cells might have an altered protease profile.

### Nischarin decreases the expression of Invadopodia proteins

**Fig. 5:**
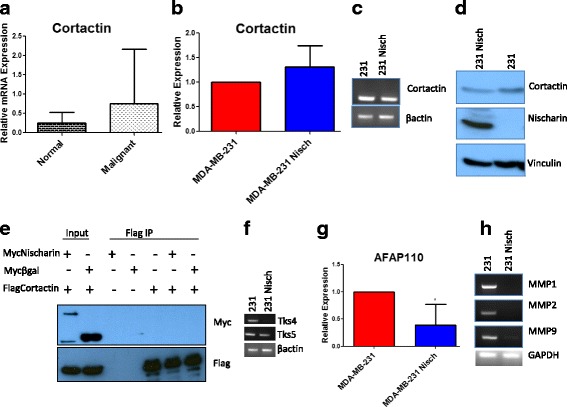
Nischarin Decreases the Expression of Invadopodia Proteins. **a** qRT-PCR of Cortactin in normal (n = 7) and malignant (*n* = 13) human breast samples. **b** qRTPCR of Cortactin in 231 (*n* = 8) and 231 Nischarin (n = 8) cells. **c** RT PCR of Cortactin RNA in 231 and 231 Nisch cells. **d** Western Blot of Cortactin protein expression in 231 and 231 Nisch cells. Vinculin is shown as a control. **e** Co-Immunoprecipitation of Myc-Nischarin and Flag-Cortactin in HEK 293 cells. **f** RT-PCR of Tks4 and Tks5 RNA in 231 and 231 Nisch cells run on a 3% agarose gel. **g** qRTPCR of AFAP110 in 231 (n = 6) and 231 Nisch (n = 8) cells. **h** RT PCR of MMPs 1, 2 and 9 RNA in 231 and 231 Nisch cells run on a 3% agarose gel. *p < 0.05, **p < 0.01

Cortactin has been shown to be good marker for invadopodia [30] and its mRNA expression is increased in malignant human breast tissues (Fig. 5a). We investigated whether Nisch regulates formation of invadopodium through Cortactin. QRT-PCR experiments showed no significant difference in the expression of Cortactin mRNA in MDA-MB-231 versus MDA-MB-231 Nisch cells (Fig. 5b). Agarose gel visualization of Cortactin cDNA from MDA-MB-231 and MDA-MB-231 Nisch cells confirmed our findings of no significant differences (Fig. 5c). Western blot detection of Cortactin also confirmed that the protein expression was not altered by the presence of Nisch (Fig. 5d). Since we did not find any changes in Cortactin RNA or protein expression, we thought Nischarin might alter invadopodia formation by interacting with Cortactin. Coimmunoprecipitation experiments demonstrated that Nischarin does not interact with Cortactin (Fig. 5e)**.** These data suggest that Nischarin uses another mechanism to prevent invadopodia formation.

Tks4 and Tks5 are adaptor proteins that are required for invadopodia formation [31]. RT-PCR of Tks4 and Tks5 showed a decrease in the expression of Tks4, but not Tks5 (Fig. 5f). During invadopodia formation, Tks5 recruits AFAP-110 to the future protrusion site [32]. We found decreased amounts of AFAP-110 in Nisch expression cells (Fig. 5g). Although the expression of, Cortactin, is not regulated by Nischarin, other invadopodium proteins seem to be affected.

Fourteen Matrix-Metallo Proteases (MMPs) have been noted to have increased expression in breast cancer tissue when compared to normal breast tissue [33]. To assess whether the presence of Nischarin in breast cancer cells affects MMPs, we performed reverse transcriptase PCR of several MMPs on MDA-MB-231 and MDA-MB-231 Nisch cells. We found reduced expression of MMPs 1, 2 and 9 in the Nischarin expressing cancer cells (Fig. 5h). Taken together, our findings suggest that the presence of Nischarin in breast cancer cells leads to a down regulation of MMPs 1, 2, and 9, which leads to a reduction in ECM degradation.

## Discussion

Epithelial-mesenchymal transition (EMT) is an important step during the process of cancer cell detachment from the primary site to the distant organs [34]. Tumor cells then use the fibril and glycoprotein rich ECM for migration and cell adhesion. Our data show the ability of the tumor suppressor, Nischarin to regulate cell attachment to the ECM. Although we have previously shown that Nisch prevents cell migration [11, 14, 23], we have not determined the exact mechanism. We confirmed that Nisch prevents cancer cell migration since we see a reduction in the percentage of wound healing.

The results from our micrographs were consistent with previous wound healing assays performed in Nisch positive and negative MDA-MB-231 cells [35]. Many cell migration assays, including the wound healing assay, are on 2D and do not take into account the complexity of the solid tumor. Since cancer cells in a solid tumor are in a three-dimensional environment, we determined Nischarin’s ability to reduce the migration of cells in 3D matrix. We identified that Nischarin was able to reduce the migration of cancer cells on 3D matrices. As cell migrates through a 3D environment, it experiences resistance from the ECM that could alter attachment, polarization and migration [36]. Of great importance to us is the effect that the 3D ECM has on FA proteins.

**Fig. 6:**
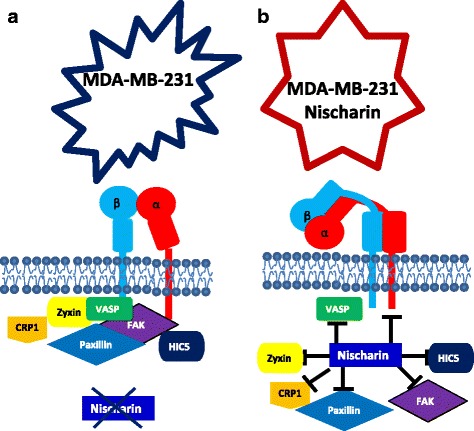
Nischarin Decreases FA Expression in Breast Cancer Cells. **a** 231 cells are able to have more FA protrusions due to the activation of the FA interactome. **b** The presence of Nisch in 231 cells inhibits the expression of key FA proteins, which results in fewer protrusions

To determine whether Nisch regulates cell-matrix attachments, we performed a screen of key FA proteins using MDA-MB-231 and MCF7 cells. Although some proteins did not have a change in expression, VASP, Paxillin, HIC-5, CRP1 and MCAM have significant differences in RNA and protein expression between Nischarin expressors and non-expressors. Reduction in FAs number per cell could possibly be due to the changes in these proteins expression. More importantly, we detected the changes were only specific to cancer cells (Fig. 6). Highly motile and invasive cancer cells already exhibit expression of oncogenic proteins that crosstalk with the FA signaling pathway. For example, cancer cells have an increase in FAK and Src activation, which contributes to increased FA protein recruitment and phosphorylation [37, 38]. This explains why introducing Nisch into non-cancerous Cos7 cells does not alter FA protein expression. Previous studies have shown that Nisch does not co-localize to FA sites [23]. No other studies have been able to explain how Nischarin reduces the expression of *ITGA5*. Since there is bidirectional crosstalk between Integrins and FA proteins, it is possible that the down regulation of Integrins leads to a down regulation of FA proteins. In other words, if there is no need for the proteins to be in FA machinery due to the unavailability of Integrins, and thus the expression of FA proteins is reduced in cancer cells.

The FA proteins form the macromolecular focal adhesion unit that helps attaching the cells to the ECM. Integrins are the proteins that actually connect the FA complexes to the ECM. We were able to see that the absence of Nisch increases the targeting of α5-Integrin to FAs (Fig. 6). Furthermore, we noticed a reduction in the amount of active α5-Integrin in Nisch-expressing breast cancer cells, suggesting a possible reason for reduction of FAs in Nisch expressing cells [11, 23].

Integrin α5β1 participates in the adhesion necessary for invadopodia formation [39], and thus it was necessary to analyze cell invasion. We have previously published cell invasion data demonstrating the ability of Nisch to reduce invasion on Fibronectin transwell inserts [11]. Our current findings showed a decrease in fluorescent matrix degradation of breast cancer cells in the presence of Nischarin. Furthermore, a greater amount of FDM degradation was seen on NIH3T3 matrices seeded with Null Nischarin MEF cells. Similarly, matrices produced from Nischarin HET MEF cells had a reduction of fiber number and width, likely due to the invasive capacity of cells with reduced Nisch expression. Transwell and gelatin degradation experiments of cell supernatants revealed that Nischarin positive cancer cells prevent degradation. The increased matrix degradation seen in cells lacking Nischarin explains why we see a reduction in fiber number and width. Our results agree with results from other groups that have shown that the formation of invadopodia protrusions correlates with ECM degradation [40].

Since we see more degradation in the Nischarin Null cells, we checked the expression of key invadopodium machinery. There was no statistical difference in Cortactin expression between Nischarin positive and negative breast cancer cells. Furthermore, Nischarin did not interact with Cortactin, the key regulator of invadopodia. We did, however, find a reduction in the key invadopodia proteins Tks4 and AFAP-110. Assessment of MMP1, 2, and 9 in the cells showed that Nischarin positive cancer cells have a reduction in expression of all three MMPs. Other studies have shown that the expression of MMPs relies on Integrin activation [41]. In fact, Integrins cooperate with MMPs to promote breast cancer cell migration and invasion [42]. We predict that Nisch indirectly affects MMP expression. When Nisch reduces Integrin expression, this leads to a downstream reduction in MMP expression. Therefore, Nischarin positive breast cancer cells have a limited ability to degrade the ECM because they have decreased expression of MMPs. Taken together; our data show that the scaffolding protein Nisch regulates cell attachment and invadopodia formation by regulating the expression of numerous proteins. These data therefore demonstrate the power of Nischarin as a tumor suppressor, and its importance in preventing cell invasion.

## Conclusions

In summary, we confirm our previous findings that Nischarin decreases cancer cell migration, and this is matrix independent. We then show that Nischarin alters the expression of other Integrins and multiple FA proteins. We further demonstrate the ability of Nisch to decrease invadopodia formation by altering the expression of some key invadopodia proteins. Since Nischarin is typically down regulated in cancer cells, these results help us understand why Nischarin-expressing cells have a slow migration rate.

## Additional file

Additional file 1: **Figure S1.** A) WT and Null MEFs were seeded onto Fibronectin coated coverslip (10 μg/ml) overnight then stained with VASP. # FA per cell and % area covered by FA was acquired using CellProfiler. B) Co-Immunoprecipitation of Myc-Nischarin and VASP in HEK293 cells. (PDF 190 kb)

## Abbreviations

ECM: Extracellular matrix
FA: Focal adhesion
MEF: Mouse embryonic fibroblast
MMP: Matrix metalloproteinase
PCR: Polymerase chain reaction
