# Supplementary figures and images for: Nischarin regulates focal adhesion and Invadopodia formation in breast cancer cells
Source: Mol Cancer. 2018 Feb 7;17:21. doi: 10.1186/s12943-018-0764-6 (PMC5803897; doi:10.1186/s12943-018-0764-6)

# Supplementary Figure 1

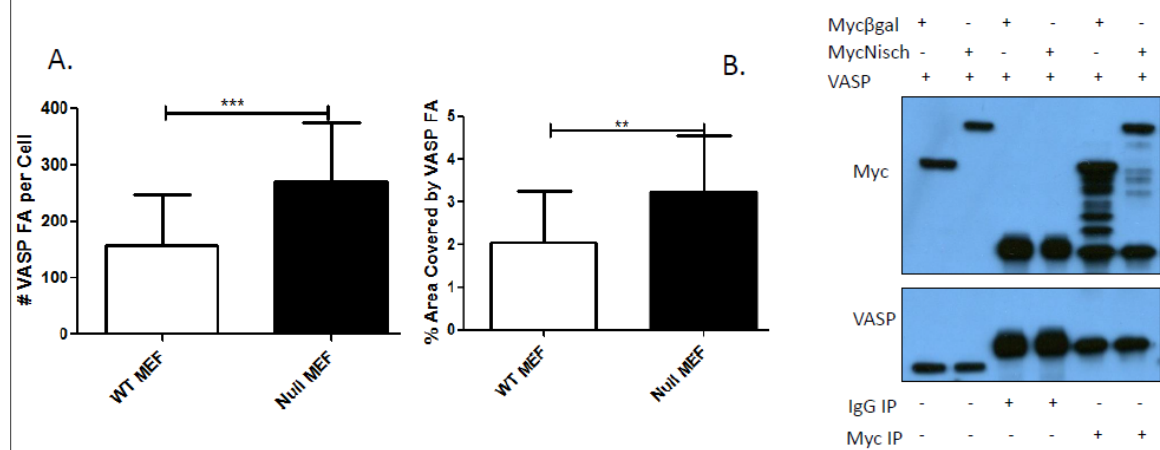

Supplement: Additional file 1: — Figure S1. A) WT and Null MEFs were seeded onto Fibronectin coated coverslip (10 μg/ml) overnight then stained with VASP. # FA per cell and % area covered by FA was acquired using CellProfiler. B) Co-Immunoprecipitation of Myc-Nischarin and VASP in HEK293 cells. (PDF 190 kb) [file 12943_2018_764_MOESM1_ESM.pdf]
